# Prognostic Indications of Elevated MCT4 and CD147 across Cancer Types: A Meta-Analysis

**DOI:** 10.1155/2015/242437

**Published:** 2015-12-08

**Authors:** Cory D. Bovenzi, James Hamilton, Patrick Tassone, Jennifer Johnson, David M. Cognetti, Adam Luginbuhl, William M. Keane, Tingting Zhan, Madalina Tuluc, Voichita Bar-Ad, Ubaldo Martinez-Outschoorn, Joseph M. Curry

**Affiliations:** ^1^Sidney Kimmel Medical College, Thomas Jefferson University, Philadelphia, PA 19107, USA; ^2^Department of Otolaryngology-Head and Neck Surgery, Thomas Jefferson University, Philadelphia, PA 19107, USA; ^3^Department of Medical Oncology, Thomas Jefferson University, Philadelphia, PA 19107, USA; ^4^Department of Pharmacology and Experimental Therapeutics, Thomas Jefferson University, Philadelphia, PA 19107, USA; ^5^Department of Pathology, Anatomy, and Cell Biology, Thomas Jefferson University, Philadelphia, PA 19107, USA; ^6^Department of Radiation Oncology, Thomas Jefferson University, Philadelphia, PA 19107, USA

## Abstract

*Background*. Metabolism in the tumor microenvironment can play a critical role in tumorigenesis and tumor aggression. Metabolic coupling may occur between tumor compartments; this phenomenon can be prognostically significant and may be conserved across tumor types. Monocarboxylate transporters (MCTs) play an integral role in cellular metabolism via lactate transport and have been implicated in metabolic synergy in tumors. The transporters MCT1 and MCT4 are regulated via expression of their chaperone, CD147.* Methods*. We conducted a meta-analysis of existing publications on the relationship between MCT1, MCT4, and CD147 expression and overall survival and disease-free survival in cancer, using hazard ratios derived via multivariate Cox regression analyses.* Results*. Increased MCT4 expressions in the tumor microenvironment, cancer cells, or stromal cells were all associated with decreased overall survival and decreased disease-free survival (*p* < 0.001 for all analyses). Increased CD147 expression in cancer cells was associated with decreased overall survival and disease-free survival (*p* < 0.0001 for both analyses). Few studies were available on MCT1 expression; MCT1 expression was not clearly associated with overall or disease-free survival.* Conclusion*. MCT4 and CD147 expression correlate with worse prognosis across many cancer types. These results warrant further investigation of these associations.

## 1. Background

*Overview of Monocarboxylate Transporters*. Monocarboxylic acids play an important role in cellular metabolism, and the regulation of this system has become a new target for understanding the pathogenesis of abnormal cellular processes such as tumorigenesis. Monocarboxylate transporters (MCTs) are 12-segment transmembrane proteins that symport protons with monocarboxylic acids through the plasma membrane [1]. These monocarboxylic acids include lactate and, to a lesser extent, pyruvate, ketone bodies, and metabolites of branched-chain amino acids. MCT family members have different characteristics regarding transport directionality and substrate specificity.

There are at least 14 members of the MCT family; all are encoded by the solute carrier gene series,* SLC16A*. Of this family, MCTs 1–4 are the best characterized thus far, with particular research emphasis placed on MCT1 and MCT4. The most studied function of MCT1 is transport of lactate into the cell, although in some physiologic conditions MCT1 can mediate lactate efflux [2]. This transporter also has a widespread expression throughout the body [2]. MCT2 has similar function to MCT1 but has a higher affinity for pyruvate and has distinct expression patterns. MCT3 expression is limited to the retinal pigment epithelium where it regulates lactate levels; its mechanism of action is not well characterized [3]. MCT4 is highly expressed in tissues dependent on glycolysis, and it plays an important role in lactate efflux from cells. MCTs 5–10 are not well characterized, although there is evidence of a role for MCT8 in targeting proteins to lysosomes and thyroid hormone transport [4]. MCT1 and MCT4 typically act as lactate importers and exporters, respectively. However, these two transporters have similar regulatory control: CD147 is a chaperone, which is essential for both MCT1 and MCT4 transport to the plasma membrane [5]. MCTs are being studied as cancer therapeutic targets since they regulate glycolytic processes via lactate transport.

**Figure 1:**
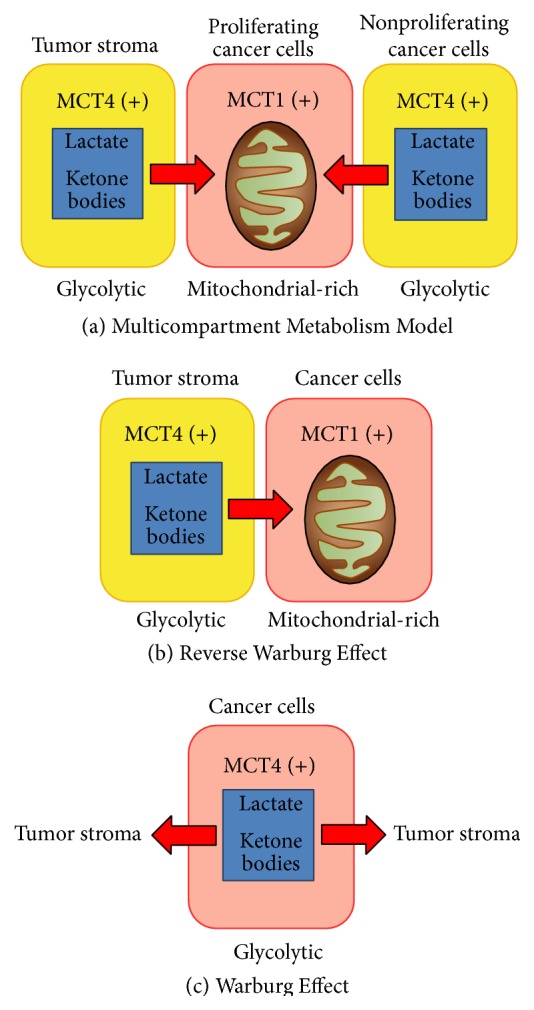
Multicompartment metabolism model in cancer. Modified with permission from Curry et al. [6].

*Cancer Metabolism and the Tumor Microenvironment*. Cancer metabolism involves a complex array of intracellular and intercellular interactions within the tumor microenvironment; understanding and intervening in these processes have allowed exploration of novel anticancer therapy approaches. A “seed and soil” hypothesis of tumor growth, which states that cancer flourishes in a favorable environment, was originally proposed by Paget [7]. Recent investigations of the metabolic microenvironment of tumors have brought this theory back to light. One of the best-known differences between cancer cell metabolism and that of healthy tissue is that tumor cells utilize glycolysis despite oxygen being present, which is termed the “Warburg Effect” ([8]; Figure 1(c)). This metabolic adaptation is postulated to confer a biosynthetic advantage for tumor development and progression due to increased carbon utilization, hypoxic adaptation, and increased rate of ATP production [9–11]. This unique glycolytic feature of tumors is the basis of fluoro-2-deoxy-glucose positron emission tomography (FDG-PET) imaging. This theory has been expanded by evidence that proliferating cancer cells may benefit from a “Reverse Warburg Effect” (Figure 1(b)) by inducing glycolysis in the surrounding tissue and deriving nutrients such as lactate from cancer-associated fibroblasts [12–14]. In a recently proposed model, the Reverse Warburg Effect is further dissected to include different populations of cancer cells: highly proliferative cancer cells and less proliferative cancer cells [6]. This Multicompartment Metabolism Model (Figure 1(a)) hypothesizes that highly proliferative cancer cells derive their lactate substrate not only from stromal cells, but also from surrounding nonproliferative cancer cells. Thus, the leading edge of the tumor with highly proliferative cancer cells takes advantage of the favorable microenvironment provided by both stroma and less proliferative cancer cells. The highly proliferative cancer cells are poorly differentiated and are believed to arise from basal stem cells, representing a group of cancer stem cells [6]. The less proliferative cancer cells have little to no expression of Ki-67, a proliferation marker; this population is also more differentiated and mitochondrially poor [6]. The tumor microenvironment is composed of proliferative cancer cells, nonproliferative cancer cells, adjacent epithelial cells, stromal cells, immune cells, and surrounding matrix. Tumor cell engraftment requires that cancer cells metabolically reprogram their microenvironment to form a suitable “nest” for tumor cell growth. This reprogramming can be explained by hydrogen peroxide secretion and HIF*α* and NF*κ*B signaling from cancer cells which induces aerobic glycolysis in surrounding tissue [12, 15]. The surrounding fibroblasts and cancer cells then are able to supply metabolic catabolites of glycolysis such as lactate and pyruvate. This “lactate shuttle” is an efficient transfer of high-energy nutrients from fibroblasts and nonproliferative cancer cells to proliferative cancer cells [6, 16, 17]. A metabolic symbiosis occurs, where fibroblasts upregulate MCT4 for lactate and ketone body export [16–18], and proliferative cancer cells import these metabolic fuels via MCT1 [16]. This type of metabolic symbiosis has been described in many different epithelial cancer types [6, 15, 19–21] and creates an environment favorable to growth, survival, and metastatic spread [13, 14]. One model proposes multiple compartments with a proliferative cancer cell population which expresses MCT1 at the tumor front with a deeper population of MCT4+ cancer cells and MCT4+ cancer-associated fibroblasts, which serve as the driving force for cancer cells to proliferate via a lactate shuttle (Figure 1, [6]).

*Monocarboxylate Transporter Expression and Cancer Prognosis*. We hypothesize that altered metabolism induces tumor progression by a similar mechanism in many cancer types that involves MCT1, MCT4, and CD147 expression. Tumoral and peritumoral expression of these three functional proteins correlate with poor prognosis in various cancers. To date, there have been no analyses correlating overall survival or disease-free survival with expression of these markers across cancer types. Though each cancer is unique, it is important to determine general oncologic principles that can be used for expansion of therapeutic trials. There are CD147 and MCT inhibitors in clinical trials in specific cancer patient populations [22, 23]. By understanding common features among different cancer types, these potential therapies can be applied more broadly.

## 2. Materials and Methods

A PubMed search for the keywords (“MCT1” OR “MCT4” OR “monocarboxylate transporters” OR “CD147” OR “EMMPRIN” OR “Basigin”) AND (“survival” OR “prognosis”) was performed. Clinical investigations into the prognostic value of MCT1, MCT4, and CD147 were selected as entries for the present study. Studies included in Forest Plot analyses were limited to those in which multivariate analysis Cox regression hazard ratio data on overall survival or disease-free survival was available. Forest Plots were constructed using RevMan 5.3 software (The Cochrane Collaboration). Forest Plot specifications were generic inverse variance for data type, fixed effect for analysis method, and hazard ratio for effect measure.

When SEM was not provided directly by the studies, they were calculated from the 95% confidence intervals by the formula SEM = (ln(Upper CI limit) − ln(Lower CI limit))/3.92 [24].

## 3. Results

### 3.1. Increased MCT4 Expression Is Associated with Decreased Overall Survival

**Figure 2:**
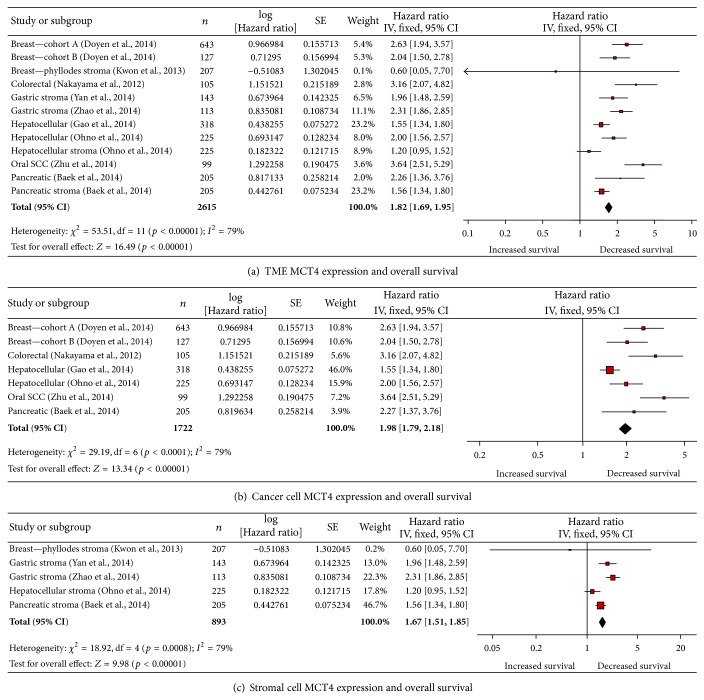
Elevated MCT4 expression is associated with decreased overall survival. (a) Elevated MCT4 expression in the tumor microenvironment is associated with decreased OS. (b) Elevated MCT4 expression in cancer cells is associated with decreased OS. (c) Elevated MCT4 expression in stromal cells is associated with decreased OS. SCC: squamous cell carcinoma.

MCT4 expression anywhere in the tumor microenvironment was associated with decreased overall survival (OS, Figure 2(a)). The 12 included studies showed that elevated MCT4 expression was associated with decreased OS by a factor of 1.82 (*p* < 0.00001, Figure 1(a)). This analysis included studies that reported either cancer cell or stromal cell MCT4 expression. Cancer types represented are pancreas (cancer and stroma) [25], breast (cancer x2) [26], phyllodes (stroma) [27], oral squamous cell carcinoma (oral SCC, cancer) [28], hepatocellular carcinoma (HCC, cancer x2, stroma) [29–31], gastric (stroma x2) [19, 20, 32], and colorectal carcinoma (CRC, cancer) [33].

High MCT4 expression specifically in cancer cells was associated with decreased OS (Figure 2(b)). The 7 included studies showed that elevated MCT4 expression was associated with decreased OS by a factor of 1.98 (*p* < 0.00001, Figure 2(b)). Cancer types included were pancreas [25], breast [26], oral SCC [28], HCC [29–31], and CRC [33]. There were 11 studies that did not have multivariate analysis data available [19, 20, 29, 30, 34–43]. Of these, 6 had statistically significant univariate analysis of elevated cancer cell MCT4 correlating with decreased OS ([29, 30, 34–36, 42, 43], see Supplementary Table 1 in Supplementary Material available online at http://dx.doi.org/10.1155/2015/242437). The other studies failed to show a significant association between elevated MCT4 and decreased OS [19, 20, 38–41].

Elevated MCT4 expression specifically by tumor-associated stroma was also associated with decreased OS (Figure 2(c)). The 5 included studies showed that elevated MCT4 expression was associated with decreased OS by a factor of 1.67 (*p* < 0.00001, Figure 2(c)). Cancer types represented are pancreas [25], phyllodes [27], gastric [19, 20, 32], and HCC [31]. There were 2 studies without multivariate analysis: one which showed no association between MCT4 expression and OS in non-small-cell lung cancer [42] and one that showed that stromal MCT4 expression correlated with decreased OS in triple-negative breast cancer under univariate analysis (*p* < 0.0001, [41]; Supplementary Table 1).

### 3.2. Increased MCT4 Expression Is Associated with Decreased Disease-Free Survival

**Figure 3:**
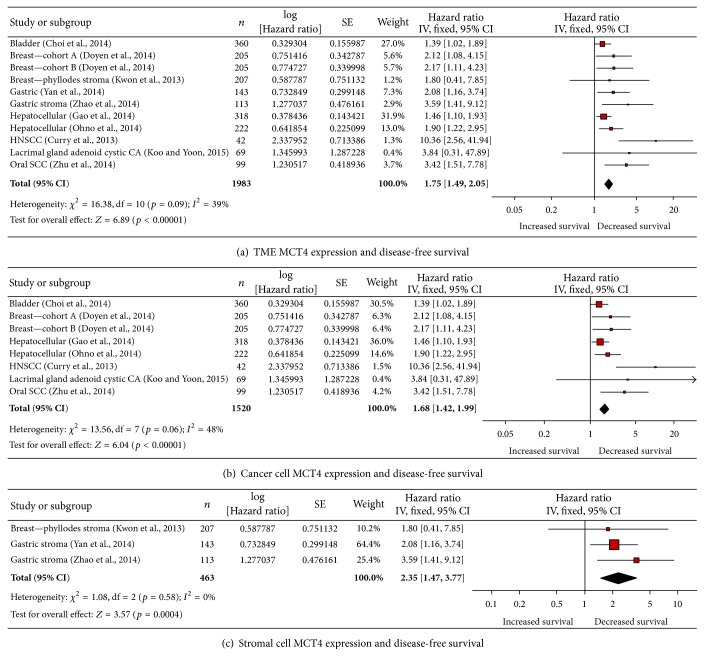
Elevated MCT4 expression is associated with decreased disease-free survival. (a) Elevated MCT4 expression in the tumor microenvironment is associated with decreased DFS. (b) Elevated MCT4 expression in cancer cells is associated with decreased DFS. (c) Elevated MCT4 expression in stromal cells is associated with decreased DFS. HNSCC: head and neck squamous cell carcinoma; CA: carcinoma; and SCC: squamous cell carcinoma.

MCT4 expression in the tumor microenvironment was associated with decreased disease-free survival (DFS, Figure 3(a)). The 11 studies included showed that elevated MCT4 expression was associated with decreased disease-free survival by a factor of 1.75 (*p* < 0.00001, Figure 3(a)). This analysis included studies that reported either cancer cell or stromal cell MCT4 expression. Cancer types represented are breast (cancer) [26], phyllodes (stroma) [27], oral SCC (cancer) [28], HCC (cancer) [29–31], gastric (cancer and stroma) [19, 20, 32], head and neck squamous cell carcinoma (HNSCC, cancer) [6], bladder (cancer) [44], and lacrimal gland adenoid cystic carcinoma (lacrimal gland ACC, cancer) [36].

Elevated MCT4 expression specifically by cancer cells was associated with decreased DFS (Figure 3(b)). The 8 included studies showed that elevated MCT4 expression was associated with decreased DFS by a factor of 1.68 (*p* < 0.00001, Figure 3(b)). Cancer types represented were breast [26], oral SCC [28], HCC [29–31], bladder [44], lacrimal gland ACC [36], and HNSCC [6]. There were 12 studies that did not have multivariate analysis data available ([19, 20, 29, 30, 34–43]; Supplementary Table 2). Of these, 6 had statistically significant univariate analysis of elevated tumoral MCT4 correlating with decreased DFS in renal cell carcinoma [34], soft tissue sarcoma [35], hepatocellular carcinoma [29, 30], Lacrimal gland adenoid cystic carcinoma [36], non-small-cell lung cancer [42], and glioblastoma multiforme [43]. The other studies did not show an association between elevated MCT4 and decreased DFS [19, 20, 37–41].

Elevated MCT4 expression in tumor-associated stroma was also associated with decreased DFS (Figure 3(c)). The 3 studies included showed that elevated MCT4 expression was associated with decreased DFS by a factor of 2.35 (*p* = 0.0004, Figure 3(c)). Cancer types represented were phyllodes [27] and gastric [19, 20, 32]. There were 2 studies without multivariate analysis available, one which showed that stromal MCT4 expression was significantly correlated with decreased DFS under univariate analysis in triple-negative breast cancer [41] and one which showed no such association in non-small-cell breast cancer ([42]; Supplementary Table 2).

### 3.3. Increased CD147 Expression in Cancer Cells Is Associated with Decreased Overall Survival

**Figure 4:**
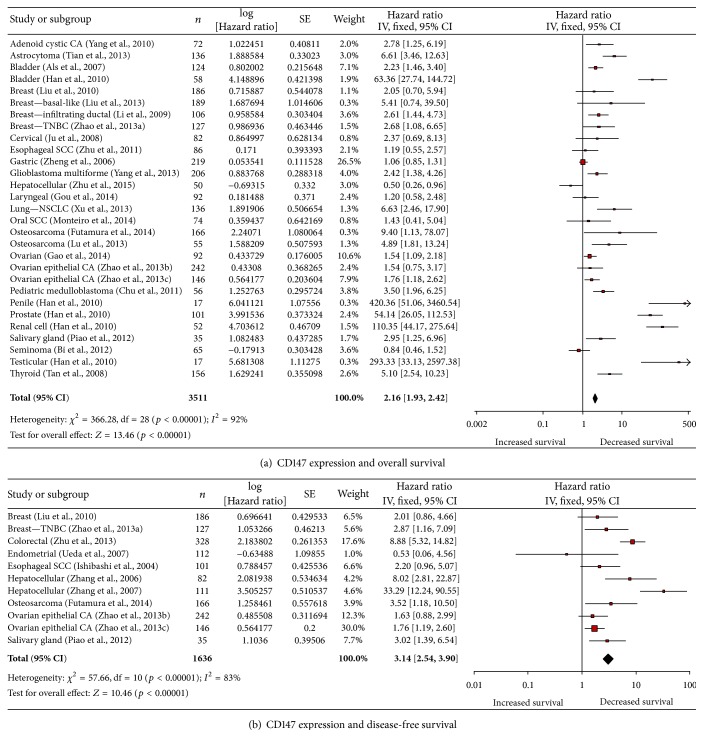
Elevated CD147 expression is associated with decreased survival. (a) Elevated CD147 expression in cancer cells is associated with decreased overall survival. (b) Elevated CD147 expression in cancer cells is associated with decreased disease-free survival. CA: carcinoma; TNBC: triple-negative breast cancer; SCC: squamous cell carcinoma; and NSCLC: non-small-cell lung cancer.

Elevated CD147 expression in cancer cells was associated with decreased OS (Figure 4(a)). The 25 included studies showed that elevated CD147 expression was associated with decreased OS by a factor of 2.16 (*p* < 0.00001, Figure 4(a)) [29, 30, 45–69]. This analysis included studies that reported only cancer cell CD147 expression. No studies had multivariate analysis of stromal CD147 expression. Of note, only 2 studies showed an increase in OS with elevated CD147 expression [57, 68].

There were 30 studies that did not have adequate multivariate analysis data available ([26, 35, 38, 44, 55–57, 66, 70–93]; Supplementary Table 3). Three of these studies had multivariate *p* values reported without the necessary hazard ratios necessary for meta-analysis [77, 78, 80]. Of these studies, 17 have statistically significant univariate analysis of elevated CD147 in cancer cells correlating with decreased OS ([44, 55–57, 66, 70–82]; Supplementary Table 3). The other studies did not show an association between elevated CD147 and OS ([26, 35, 38, 83–93]; Supplementary Table 3).

### 3.4. Elevated CD147 Expression in Cancer Cells Is Associated with Decreased Disease-Free Survival

Elevated CD147 expression in cancer cells was associated with decreased DFS (Figure 4(b)). The 11 included studies showed that elevated CD147 expression was associated with decreased DFS by a factor of 3.14 (*p* < 0.00001, Figure 4(b)). This analysis included studies that reported only cancer cell CD147 expression. No studies reported had multivariate analysis of stromal CD147 expression and survival. Cancer types represented include esophageal SCC [94], salivary gland cancer [67], breast cancer [48], triple-negative breast cancer [51, 63, 64], osteosarcoma [61], colorectal cancer [95], ovarian epithelial cancer [63, 64], endometrial cancer [96], and hepatocellular carcinoma [97, 98]. Of note, only one study showed an increase in DFS with elevated CD147 expression [96]. There were 13 studies that did not have multivariate analysis data available ([26, 57, 60, 62, 71, 72, 77, 82, 84, 91, 92, 99, 100]; Supplementary Table 4). Of these, 8 had statistically significant univariate analysis revealing that elevated CD147 correlates with decreased DFS ([26, 57, 62, 71, 72, 82, 99, 100]; Supplementary Table 4). The other studies showed no association between elevated MCT4 and DFS ([60, 77, 84, 91, 92]; Supplementary Table 4).

### 3.5. MCT1 Expression and Prognosis

There is currently insufficient high-quality data available to conduct a meta-analysis of studies examining the correlation of OS or DFS with cancer cell expression of MCT1. However, a review of the literature on MCT1's impact on cancer prognosis is provided here.

**Figure 5:**
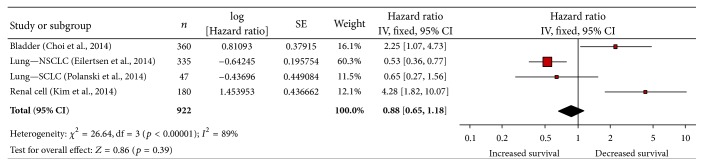
MCT1 expression in the tumor microenvironment is not associated with overall survival. NSCLC: non-small-cell lung cancer; SCLC: small-cell lung cancer.

Multivariate analysis on MCT1 expression and OS was only available in 4 studies (Figure 5). Increased MCT1 expression in cancer cells was associated with decreased OS in bladder cancer and renal cell carcinoma [34, 44]. Increased MCT1 expression was shown to either increase or have no effect on OS in NSCLC and SCLC [39, 42].

An additional five studies that analyzed MCT1 expression and OS did not have multivariate analysis data available ([19, 20, 25, 38, 71, 101]; Supplementary Table 5). Of these, only 2 studies showed a significant decrease in OS associated with elevated MCT1 expression (*p* = 0.021, [35]; *p* = 0.014, [19, 20]). The remainder of the studies failed to show a statistically significant change in survival associated with MCT1 expression ([25, 38, 71]; Supplementary Table 5). A single study evaluated elevated MCT1 expression and DFS in bladder cancer, but this univariate analysis failed to show a significant association (*p* = 0.065, [71]).

Interestingly, only one study examined cancer and stromal cell expression of MCT1 individually. In a study of 335 cases of NSCLC, univariate analysis revealed that increased MCT1 expression in stromal cells corresponded significantly with poor disease-specific survival (*p* = 0.003), but increased MCT1 expression in cancer cells corresponded with a favorable DFS (*p* = 0.020). Both these associations held in multivariate analysis (*p* = 0.001, 0.016, resp., [42]).

## 4. Discussion

The “Reverse Warburg” model of tumor metabolism hypothesizes a compartmentalized metabolic tumor microenvironment. The transfer of molecules between compartments allows for highly proliferative cancer cells to maintain oxidative phosphorylation while CAFs and less proliferative cancer cells provide metabolic fuels generated by glycolysis. The monocarboxylate transporter system allows the intercellular exchange of metabolites that fuel different tumoral compartments. In particular, MCT1 and MCT4 play crucial roles in the influx and efflux, respectively, of lactate, pyruvate, and other metabolites. CD147 serves as a chaperone for MCT1 and MCT4 and is essential in their expression [5]. MCT1, MCT4, and CD147 are functional biomarkers for metabolic compartmentalization in cancer, and their presence has implications for tumor aggressiveness and prognosis.

The monocarboxylate transporter system has been studied in various cancer types, and here we show that the association between MCT1, MCT4, and CD147 is similar across many types of cancer. This is the first study to investigate the significance of these biomarkers across such varied types of cancer, and, although each cancer is biologically unique, the data presented here suggests that tight metabolic coupling with catabolite transfer between different tumor cells is associated with outcomes. Cancer cells often exploit previously existing cellular functions in order to fuel their own growth; a system of energy transfer may play a role in promoting tumorigenesis in many types of cancer, just as TP53 mutations have been shown to promote growth and suppress apoptosis in many cancers. We provide evidence that expression of MCT4 and CD147 predicts clinical behavior in many different cancers, even if their particular role in each type of cancer is not yet well described. To date, there have been few studies examining the MCT system across cancer types, and none which examine the breadth of cancer types were analyzed in this study. The reviews that cover this subject have been limited to the molecular mechanisms of lactate transporters in tumor metabolism [102, 103]. The current study bolsters the external validity of studies on expression of MCT4 and CD147 and prognosis.

In determining the impact of MCT1, MCT4, and CD147 expression on outcomes, attention must be paid to expression levels as well as expression patterns. In the Multicompartment Metabolism Model, a highly proliferative population of cancer cells express MCT1 strongly, and, in fact, much stronger than the less proliferative cancer cells and stromal cells around them. In contrast, these less proliferative cancer cells express MCT4 strongly, while MCT4 expression in highly proliferative cancer cells is low. Thus, the reported location of MCT4 staining is important when considering its effect on tumor biology and prognosis. There are differences in transporter expression in cancer cells in the tumor leading edge versus other cancer cells and stromal cells. The specific combined expression pattern of MCT1 in cancer cells and MCT4 in stromal cells was associated with decreased DFS in prostate cancer [104, 105]. Expression patterns and colocalization of MCT1, MCT4, and CD147 are also discussed in breast cancer, ovarian cancer, colorectal cancer, and lung cancer [106]. Understanding the similarities and differences of these patterns across cancer types indicates their significance for prognosis.

This meta-analysis highlights the association between stromal cells and aggressive cancer. Tumor stroma is composed of cancer-associated fibroblasts (CAFs), infiltrating immune cells, and angiogenic vascular cells. The studies in this analysis use various definitions of stromal cells and include stroma between cancer cells and stroma surrounding foci of cancer cells. These studies typically do not differentiate between CAFs and other stromal components, but, in general, define cancer associated stroma as noncancerous cells in proximity to cancer cells. Our results corroborate the recent literature asserting the importance of stromal cells in carcinogenesis, specifically by altering cellular energetics. CAFs have been implicated in carcinogenesis by sustaining proliferative signaling, evading growth suppression, avoiding immune destruction, activating invasion, inducing angiogenesis, resisting cell death, and deregulating cellular energetics [107]. CAFs are also thought to detoxify the tumor microenvironment and provide nutrients to cancer cells [108]. Some researchers have also found that cancer cells produce reactive oxygen species, which influences CAFs to undergo mitophagy and switch to glycolytic metabolism [109]. The role of CAFs in cancer progression and as a therapeutic target is being studied extensively.

Current models of cancer metabolism attempt to encompass not only cancer cells but also the local environment, including the surrounding stromal cells and extracellular matrix. There are a wide array of metabolic changes that occur during carcinogenesis involving a complex coordination between intracellular and intercellular pathways [110], with mitochondrial metabolism changes at the hub of many of these alterations [111]. The interactions between these compartments are fundamental in understanding carcinogenesis and cancer progression. The Warburg Effect (Figure 1(a)) posits that cancer cells utilize glycolysis despite the presence of oxygen and export the lactate produced into the surrounding environment. The Reverse Warburg Effect (Figure 1(b)) describes a metabolic interaction between cancer and stromal cells where glycolysis performed by stromal cells produces lactate which is then shuttled via a monocarboxylate transport system to cancer cells which then have an ample fuel supply to produce energy via oxidative phosphorylation. A newer model, termed the Multicompartment Metabolism Model (Figure 1(c)), is described similarly to the Reverse Warburg Effect; however, it divides the cancer cell compartment into a highly proliferative population and a relatively less proliferative population. Stromal and cancer cells with low proliferation rates provide nutrients for proliferative cancer cells in the Multicompartment Metabolism Model.

The evidence in this meta-analysis supports the Multicompartment Metabolism Model as MCT4 and CD147 expression decreased survival in all scenarios, whether the increased expression levels were found in cancer cells or stromal cells. The decreased survival rates associated with increased MCT4 expression in cancer cells are not fully explained by the Reverse Warburg Effect as this model would lead one to expect that only stromal cell MCT4 expression could contribute to cancer progression. Some studies which associate increased MCT4 expression in cancer cells with decreased survival using the Warburg Effect as a model attribute decreased survival to an acidic microenvironment [26, 29–31, 33] provided by MCT4-mediated lactate efflux causing matrix metalloproteinase activation [29, 30], cathepsin activation [29, 30], decreased natural killer cell activation [29, 30], decreased effectiveness of chemotherapy [26], and increased integrin interactions [33]. Another proposed mechanism which is in concert with the Warburg Effect is the activation of AKT and MEK-ERK pathways in cancer cells with increased MCT4 expression contributing to cancer progression [28–30]. While microenvironment acidification and downstream intracellular pathways may play a role in tumor progression with cancer cell MCT4 expression, we submit that the lactate efflux has additional effects through providing substrates which aid proliferative cancer cell populations. In fact, some studies on the Warburg Effect suggest that cancer cell MCT4 expression may provide enrichment to cancer stem cells [26, 29, 30] and Choi et al. mention that peripheral tumor cells may import this lactate via MCT1 [44]. These descriptions are in line with the Multicompartment Metabolism Model in which MCT4+ nonproliferative cancer cells provide substrates for proliferative MCT1+ cancer cells. There is further evidence that lactate catabolism in cancer may involve MCT1, with lactate uptake specific to aerobic tumor regions [112]. The studies provided are heterogeneous in nature, and hence it is possible that multiple types of metabolism models are found in the different cancer types and even between different areas within a single tumor.

As more is discovered about MCT1, MCT4, and CD147 as functional biomarkers, they become attractive targets for anticancer therapies. Many cancers become increasingly drug-resistant as therapies are initiated and continued, and these new therapeutic targets could prove invaluable in improving patient outcomes [51, 63, 64]. In fact, CD147 coexpression with MCT1 or MCT4 is associated with increased likelihood of multidrug resistance markers [100]. Currently, the main targets for such pharmacologic intervention are the family of monocarboxylate transporters and their regulatory proteins.

A recent study by Amorim et al. addresses the effects of lactate transport inhibition in human colorectal cancer cell lines using the compounds *α*-cyano-4-hydroxycinnamate (CHC), DIDS (a stilbene derivative), and quercetin, a bioflavonoid, which are known to inhibit lactate transport. They demonstrated that MCT activity inhibition inhibited CRC cells biomass in a dose-dependent manner, increased cell death and decreased cell proliferation, and potentiated the cytotoxicity of 5-fluorouracil in CRC cells pretreated with the MCT inhibitors [113]. However, historically, MCT inhibitors have lacked specificity; *α*-cyano-4-hydroxycinnamate (CHC), stilbene disulfonates, phloretin, quercetin, and organomercurial reagents were often more potent at inhibiting other cellular functions than plasma membrane lactate transport. More recently, however, new high-affinity MCT inhibitors have been developed and are being investigated both* in vitro* and* in vivo* as anticancer agents.

Draoui et al. investigated 7-aminocarboxycoumarin (7ACC) in xenograft models of cervical, breast, and bladder cancers [114]. 7ACC inhibits lactate influx but not efflux in cells expressing MCT1 and MCT4; in cancer types that express MCT1 and MCT4, 7ACC decreased xenograft tumor growth. In prostate cancer research, AR-C155858, an inhibitor of MCT1 and MCT2, has been shown to result in a significant decrease in proliferation and increased apoptosis in murine tumor tissues with no significant effect on benign tissue [115]. Currently, AZD3965, which is an orally administrable second-generation MCT1/MCT2 inhibitor, is being investigated in a Phase I clinical trial for the treatment of advanced solid tumors, particularly prostate cancer, gastric cancer, and diffuse large B cell lymphoma [23].

Metformin and other biguanides have much cross-reactivity with the lactate transport system as oxidative phosphorylation inhibitors. These biguanides have received much attention in anticancer therapy recently and have been shown to have a synergistic anticancer effect when combined with inhibition of MCT1, MCT4, or CD147 [116–118]. There are currently many clinical trials evaluating the effect of metformin on cancer progression. One study is specifically evaluating whether metformin can interrupt the metabolic coupling between stroma and epithelial cancer cells in head and neck squamous cell carcinoma [119].

MCT4 is a promising target for cancer pharmacotherapy, but there is no published data on specific MCT4 inhibitors to date. There is currently a Small Business Innovation Research Grant awarded to Vettore LLC to develop such an inhibitor [120]. Other agents which decrease MCT4 levels, such as siRNA [28, 37], shRNA [43], and N-acetylcysteine [17], have shown promise in decreasing MCT4 and are being studied as anticancer therapies.

CD147 has been evaluated as a therapeutic target most extensively in hepatocellular carcinoma. Metuximab, a monoclonal antibody specific to CD147, has been shown to decrease HCC recurrence after liver transplantation [121] or radiofrequency ablation [122] and increased OS in HCC patients when combined with chemoembolization [123, 124]. Metuximab is being currently studied in a clinical trial to assess its efficacy in preventing HCC recurrence [22]. Anti-CD147 antibodies have also shown promise in an* ex vivo* HNSCC model [125]. Other cancer types, such as oral SCC [126], HNSCC [127], pancreatic cancer [128], melanoma [129], and colorectal carcinoma [86, 87], have shown to be affected by CD147 levels* in vitro* and* in vivo*; however, these results have not resulted in clinical trials to date.

While our data show that both increased MCT4 expression in the tumor microenvironment, stroma, or cancer and increased CD147 expression in cancer cells are both associated with decreased OS and DFS, our analysis is limited by the fact that studies that do not demonstrate statistical significance are less likely to have published data and that some studies are not amenable to further statistical analysis. Indeed several papers reported associations between the studied biomarkers and OS or DFS simply as “not significant.”

Another limitation is the lack of uniformity in the calculation of positivity of biomarker expression. There was significant variation in the methodology by which the included studies designated specimens as positive for a given marker—some using a binary system and others grading along a spectrum. Additionally, characterization of the intensity and density of immunohistochemical staining was also subject to variability, with some studies using a computed algorithm and others relying on the graded observations of one or several pathologists.

An additional limitation is that weighted hazard ratios cannot be compared due to heterogeneity of the data. For example, we cannot assess whether MCT4 has a greater prognostic value when high expression is found in cancer cells versus stromal cells.

Looking to future studies, the tumor microenvironment metabolism will be better understood as more data on both cancer cell and stromal cell marker expression become available. Further investigation into the interaction among these biomarkers in the tumor microenvironment will be necessary to better qualify them as therapeutic targets. For example, CD147 has multiple potential mechanisms of actions to induce cancer aggressiveness. For example, CD147 increases angiogenesis via upregulation of VEGF and metalloproteinases [130, 131], increased EGFR expression [127], and increased invasion and metastasis via MMP upregulation [132]. However, multiple studies have shown cancer-modifying behaviors of CD147 are intricately related with expression of MCT1 and MCT4 [86, 87, 128]. Additionally, cytoplasmic versus membranous CD147 expression may complicate the prognostic effects of this protein [85, 92].

In conclusion, this meta-analysis of published studies identifies elevated MCT4 and CD147 as poor prognostic biomarkers across many cancers. The potential to exploit these findings to develop novel, effective treatments warrants more large-scale and standardized investigations.

## Supplementary Material

Data obtained via univariate analysis concerning MCT1, MCT4, and CD147 expression patterns in relation to overall and disease-free survival. This information is presented separately since only data obtained via multivariate analysis have been included in the Forest Plot meta-analyses.
Supplementary Table 1: Elevated MCT4 expression is associated with decreased overall survival: studies without multivariate analysis provided (HCC: hepatocellular carcinoma, TACE: transarterial chemoembolization, ACC: adenoid cystic carcinoma, SCC: squamous cell carcinoma, TNBC: triple-negative breast cancer, and NSCLC: non-small cell lung cancer).
Supplementary Table 2: Elevated MCT4 expression is associated with decreased disease-free survival: studies without multivariate analysis provided (CA: carcinoma).
Supplementary Table 3: Elevated CD147 expression is associated with decreased overall survival: studies without multivariate analysis provided.
Supplementary Table 4: Elevated CD147 expression is associated with decreased disease-free survival: studies without multivariate analysis provided.
Supplementary Table 5: MCT1 expression is not associated with prognosis: studies without multivariate analysis provided (OS: overall survival and DFS: disease-free survival).
