# Supplementary material for: Prognostic Indications of Elevated MCT4 and CD147 across Cancer Types: A Meta-Analysis
Source: Biomed Res Int. 2015 Dec 8;2015:242437. doi: 10.1155/2015/242437 (PMC4686628; doi:10.1155/2015/242437)
Supplement: Supplementary file 1 — Data obtained via univariate analysis concerning MCT1, MCT4, and CD147 expression patterns in relation to overall and disease-free survival. This information is presented separately since only data obtained via multivariate analysis have been included in the Forest Plot meta-analyses. Supplementary Table 1: Elevated MCT4 expression is associated with decreased overall survival: studies without multivariate analysis provided (HCC: hepatocellular carcinoma, TACE: transarterial chemoembolization, ACC: adenoid cystic carcinoma, SCC: squamous cell carcinoma, TNBC: triple-negative breast cancer, and NSCLC: non-small cell lung cancer). Supplementary Table 2: Elevated MCT4 expression is associated with decreased disease-free survival: studies without multivariate analysis provided (CA: carcinoma). Supplementary Table 3: Elevated CD147 expression is associated with decreased overall survival: studies without multivariate analysis provided. Supplementary Table 4: Elevated CD147 expression is associated with decreased disease-free survival: studies without multivariate analysis provided. Supplementary Table 5: MCT1 expression is not associated with prognosis: studies without multivariate analysis provided (OS: overall survival and DFS: disease-free survival). [file 242437.f1.docx]

| **Author** | **Cancer Type** | **Expression Location** | **n** | **Univariate Analysis p-value** |
| --- | --- | --- | --- | --- |
| Witkiewicz et al., 2012 | TNBC | Stroma | 159 | <0.0001 |
| Pinheiro et al., 2014 | Soft Tissue Sarcoma | Cancer | 48 | 0.003 |
| Kim et al., 2015 | Renal Cell Carcinoma | Cancer | 190 | 0.016 |
| Gao et al., 2014 | HCC (after TACE) | Cancer | 318 | 0.02 |
| Koo et al., 2015 | Lacrimal Gland ACC | Cancer | 75 | 0.027 |
| Eilertsen et al., 2014 | NSCLC | Cancer | 335 | 0.027 |
| Lim et al., 2014 | Glioblastoma Multiforme | Cancer | N/A | <0.05 |
| Eilertsen et al., 2014 | NSCLC | Stroma | 335 | 0.11 |
| Sweeny et al., 2012 | Cutaneous SCC | Cancer | 100 | 0.15 |
| Meijer et al., 2012 | NSCLC | Cancer | 84 | 0.191 |
| Gerlinger et al., 2012 | Renal Cell Carcinoma | Cancer | 50 | 0.22 |
| Polanski et al., 2014 | Small Cell Lung Cancer | Cancer | 50 | 0.574 |
| Zhao et al., 2014 | Gastric Cancer | Cancer | 113 | 0.828 |
| Witkiewicz et al., 2012 | TNBC | Cancer | 159 | 0.97 |

Supplementary Table 1. Elevated MCT4 expression is associated with decreased overall survival: studies without multivariate analysis provided. HCC, hepatocellular carcinoma; TACE, transarterial chemoembolization; ACC, adenoid cystic carcinoma; SCC, squamous cell carcinoma; TNBC, triple-negative breast cancer; NSCLC, non-small cell lung cancer.

| **Author** | **Cancer Type** | **Expression Location** | **n** | **Univariate Analysis p-value** |
| --- | --- | --- | --- | --- |
| Witkiewicz et al., 2012 | TNBC | Stroma | 159 | <0.0001 |
| Pinheiro et al., 2014 | Soft Tissue Sarcoma | Cancer | 48 | 0.003 |
| Kim et al., 2015 | Renal Cell Carcinoma | Cancer | 190 | 0.016 |
| Gao et al., 2014 | HCC (after TACE) | Cancer | 318 | 0.02 |
| Koo et al., 2015 | Lacrimal Gland ACC | Cancer | 75 | 0.027 |
| Eilertsen et al., 2014 | NSCLC | Cancer | 335 | 0.027 |
| Lim et al., 2014 | Glioblastoma Multiforme | Cancer | N/A | <0.05 |
| Eilertsen et al., 2014 | NSCLC | Stroma | 335 | 0.11 |
| Sweeny et al., 2012 | Cutaneous SCC | Cancer | 100 | 0.15 |
| Meijer et al., 2012 | NSCLC | Cancer | 84 | 0.191 |
| Gerlinger et al., 2012 | Renal Cell Carcinoma | Cancer | 50 | 0.22 |
| Polanski et al., 2014 | Small Cell Lung Cancer | Cancer | 50 | 0.574 |
| Zhao et al., 2014 | Gastric Cancer | Cancer | 113 | 0.828 |
| Witkiewicz et al., 2012 | TNBC | Cancer | 159 | 0.97 |

Supplementary Table 2. Elevated MCT4 expression is associated with decreased disease-free survival: studies without multivariate analysis provided. CA, carcinoma.

| **Author** | **Cancer Type** | **n** | **Univariate Analysis p-value** |
| --- | --- | --- | --- |
| Kaira et al., 2015 | Pancreatic Cancer | 97 | 0.0003 |
| Huang et al., 2009 | Tongue SCC | 68 | <0.001 |
| Nakamura et al., 2012 | Endometrial Carcinoma | 134 | <0.001 |
| Wang et al., 2011 | Nasopharyngeal Carcinoma | 69 | 0001 |
| Zhu et al., 2015 | Epithelial Ovarian Carcinoma | 92 | 0.002 |
| Du et al., 2009 | Nasopharyngeal Carcinoma | 197 | 0.003 |
| Han et al., 2010 | Prostate Cancer | 101 | <0.01 |
| Liang et al., 2009 | Renal Cell Carcinoma | 53 | <0.01 |
| Davidson et al., 2003 | Serous Ovarian Carcinoma | 69 | 0.012 |
| Afonso et al., 2014 | Bladder Cancer | 114 | 0.018 |
| Kim et al., 2014 | Renal Cell Carcinoma | 180 | 0.02 |
| Choi et al., 2014 | Bladder Cancer | 360 | 0.029 |
| Huang et al., 2012 | Tongue SCC | 80 | 0.029 |
| Yu et al., 2015 | Oral Tongue SCC | 32 | 0.0452 |
| Liu et al., 2009 | Non-Hodgkin’s Lymphoma | 62 | <0.05 |
| Wan et al., 2012 | Esophageal SCC | 52 | <0.05 |
| Yang et al., 2013 | Hypopharyngeal Carcinoma | 101 | <0.05 |
| Sillanpää et al., 2007 | Epithelial Ovarian Carcinoma | 362 | >0.05 |
| Pinheiro et al., 2014 | Soft Tissue Sarcoma | 86 | 0.067 |
| Buergy et al., 2009 | Colorectal Cancer | 40 | 0.091 |
| Sweeny et al., 2012 | Cutaneous SCC | 44 | 0.17 |
| Doyen et al., 2014 | Breast Cancer | 127 | 0.18 |
| Rosenthal et al., 2003 | Laryngeal SCC | 33 | 0.18 |
| Tsai et al., 2007 | Pancreatic Cancer | 70 | 0.189 |
| Tsai et al., 2006 | Hepatocellular Carcinoma | 40 | 0.35 |
| Zeng et al., 2011 | Non-Small Cell Lung Cancer | 66 | 0.431 |
| Li et al., 2005 | Hepatocellular Carcinoma | 51 | 0.476 |
| Sienel et al., 2008 | Lung Adenocarcinoma | 57 | 0.6 |
| Tsai et al., 2013 | Astrocytoma | 77 | 0.7274 |
| Sienel et al, 2008 | Lung SCC | 56 | 0.79 |
| Li et al., 2013 | Pancreatic Cancer | 80 | 1 |

Supplementary Table 3. Elevated CD147 expression is associated with decreased overall survival: studies without multivariate analysis provided.

| **Author** | **Cancer Type** | **n** | **Univariate Analysis p-value** |
| --- | --- | --- | --- |
| Nakamura et al., 2012 | Endometrial Cancer | 134 | <0.001 |
| Huang et al., 2014 | Cervical SCC | 132 | <0.001 |
| Kaira et al., 2015 | Pancreatic Cancer | 97 | 0.0042 |
| Chen et al., 2010 | Epithelial Ovarian Cancer | 120 | 0.009 |
| Zhu et al., 2015 | Epithelial Ovarian Cancer | 92 | 0.014 |
| Afonso et al., 2014 | Bladder Cancer | 114 | 0.027 |
| Lu et al., 2013 | Osteosarcoma | 55 | <0.05 |
| Doyen et al., 2014 | Breast Cancer | 127 | 0.05 |
| Sillanpää et al., 2007 | Epithelial Ovarian Cancer | 362 | >0.05 |
| Li et al., 2005 | Hepatocellular Carcinoma | 51 | 0.087 |
| Yu et al., 2015 | Oral Tongue SCC | 32 | 0.0916 |
| Buergy et al., 2009 | Colorectal Cancer | 40 | 0.096 |
| Monteiro et al., 2014 | Oral SCC | 74 | 0.882 |

Supplementary Table 4. Elevated CD147 expression is associated with decreased disease-free survival: studies without multivariate analysis provided.

| **Author** | **Cancer Type** | **n** | **Univariate Analysis p-value** |
| --- | --- | --- | --- |
| Zhao et al., 2014 | Osteosarcoma | 61 | 0.014 (OS) |
| Pinheiro et al., 2013 | Soft Tissue Sarcoma | 86 | 0.021 (OS) |
| Afonso et al., 2014 | Bladder Cancer | 114 | 0.053 (DFS) |
| Afonso et al., 2014 | Bladder Cancer | 114 | 0.065 (OS) |
| Sweeny et al., 2012 | Cutaneous SCC | 44 | 0.11 (OS) |
| Baek et al., 2014 | Pancreatic Cancer | 205 | 0.273 (OS) |

Supplementary Table 5. MCT1 expression is not associated with prognosis: studies without multivariate analysis provided. OS, overall survival; DFS, disease-free survival.
